# Achieving universal health coverage and sustainable development goals by 2030: investment estimates to increase production of health professionals in India

**DOI:** 10.1186/s12960-023-00802-y

**Published:** 2023-03-02

**Authors:** Anup Karan, Himanshu Negandhi, Mehnaz Kabeer, Tomas Zapata, Dilip Mairembam, Hilde De Graeve, James Buchan, Sanjay Zodpey

**Affiliations:** 1 Indian Institute of Public Health-Delhi, Public Health Foundation of India, Plot No. 47, Sector 44, Institutional Area, Gurugram, Haryana 122002 India; 2 South-East Asia Regional Office, World Health Organization, Indraprastha Estate, Mahatma Gandhi Marg, Outer Ring Road, New Delhi, 110002 India; 3 World Health Organization, India Office, RK Khanna Tennis Stadium, Africa Avenue, New Delhi, 110 029 India; 4 Faculty of Health, WHO Collaborating Centre, University of Technology, Sydney, Australia

**Keywords:** Health workforce, Human resources for health, Investment in health, India

## Abstract

**Background:**

COVID-19 has reinforced the importance of having a sufficient, well-distributed and competent health workforce. In addition to improving health outcomes, increased investment in health has the potential to generate employment, increase labour productivity and foster economic growth. We estimate the required investment for increasing the production of the health workforce in India for achieving the UHC/SDGs.

**Methods:**

We used data from National Health Workforce Account 2018, Periodic Labour Force Survey 2018–19, population projection of Census of India, and government documents and reports. We distinguish between total stock of health professionals and active health workforce. We estimated current shortages in the health workforce using WHO and ILO recommended health worker:population ratio thresholds and extrapolated the supply of health workforce till 2030, using a range of scenarios of production of doctors and nurses/midwives. Using unit costs of opening a new medical college/nursing institute, we estimated the required levels of investment to bridge the potential gap in the health workforce.

**Results:**

To meet the threshold of 34.5 skilled health workers per 10 000 population, there will be a shortfall of 0.16 million doctors and 0.65 million nurses/midwives in the total stock and 0.57 million doctors and 1.98 million nurses/midwives in active health workforce by the year 2030. The shortages are higher when compared with a higher threshold of 44.5 health workers per 10 000 population. The estimated investment for the required increase in the production of health workforce ranges from INR 523 billion to 2 580 billion for doctors and INR 1 096 billion for nurses/midwives. Such investment during 2021–2025 has the potential of an additional employment generation within the health sector to the tune of 5.4 million and to contribute to national income to the extent of INR 3 429 billion annually.

**Conclusion:**

India needs to significantly increase the production of doctors and nurses/midwives through investing in opening up new medical colleges. Nursing sector should be prioritized to encourage talents to join nursing profession and provide quality education. India needs to set up a benchmark for skill-mix ratio and provide attractive employment opportunities in the health sector to increase the demand and absorb the new graduates.

**Supplementary Information:**

The online version contains supplementary material available at 10.1186/s12960-023-00802-y.

## Introduction

Strengthening human resources for health (HRH) is a prerequisite to a strong and resilient health system. Only by securing a sufficient, equitably distributed, adequately supported and well-performing health workforce can a country meet its health goals [1, 2]. COVID-19 has reinforced the criticality of this. However, several studies in the recent past have highlighted global shortage and skewed distribution of HRH and the need for increased investment in HRH at the global level [1–4]. The Global Strategy on Human Resources for Health: Workforce 2030 projected a shortfall of 18 million health workers by 2030, mostly in low- and lower-middle-income countries [4–6]. A recent WHO report estimated a shortage of about 1.8 million health workforce in India alone [7]. Given the acute shortage of health workforce coupled with the current health crisis posed by COVID-19 pandemic, India needs to significantly enhance investment in health in general and HRH in particular. The present study aims to estimate the potential shortages of health workforce in India by the terminal year of the Sustainable Development Goals (SDGs) and a required level of investment for increased production of health workforce to bridge this gap.

United Nations High-Level Commission on Health Employment and Economic Growth (ComHEEG), noted that enhanced investment in health workforce can deliver a triple return of improved health outcomes, global health security and economic growth [8]. The Global Strategy Report also emphasized that investment in health workforce is a driver of progress towards several SDGs [4, 8].

The current COVID-19 pandemic significantly exposed the limitations of India’s health system. With the highest number of COVID-19 infections (over 43 million) and the case fatality (approximately 521 thousand), the need for increased investment in India’s health system is the most important learning from the current pandemic crisis and needs urgent policy attention.

Increased investment in health in India is a long felt-need. India’s National Health Policy 2017 set a spending target of 2.5% of GDP by 2025, from a current level of 1.1% of the GDP [9]. The 15th Finance Commission and the *NITI Aayog* have also emphasized the need for enhanced government health spending. The health sector in India, currently growing at more than 22% per annum, is likely to grow to US$ 372 billion by 2022 and provides an opportunity for increased investment in several areas of health sector [10].

Theoretically, investments in health and population health are positively linked, despite the link between the two being influenced by a host of socio-economic and contextual factors [2]. Accordingly, an enhanced investment in HRH, to improve HRH availability (both quantitatively and qualitatively), improves population health and fosters economic growth through various channels including pathways of a strong health system, improved equity in access to healthcare by population, increased employment generation and labour productivity [8]. However, the extent to which investment in HRH will influence population health, labour productivity and economic growth will crucially depend on a productively employed health workforce instead of just increased production of health professionals. If migration and other labour market attrition of health workforce are high and demand for health workforce is low, the benefits of increased investment in HRH will be smaller compared to the usual potential of such investments. The return on investment in HRH can be maximized by reducing health labour market attrition. and increasing demand for health workforce.

Investment in health workforce in India has the potential to advance multiple development priorities within and beyond health sectors. Enhanced investment in the health workforce in India will not only strengthen the health system and improve the accessibility to health workers but also generate employment opportunities for health professionals, associate health workers and subordinate/support staff and other junior health workers, enhance female labour force participation and share of formal employment in total employment [7]. More than 90% of employment in India is in the informal sector. However, in the health sector, formal employment is as high as 50%. Also, increasing the number of health jobs is a major opportunity to rapidly increase the participation of women in the labour market [7, 11, 12].

Several studies in the recent past have highlighted acute shortage, skewed distribution and poor quality of health workforce in India [12–19] and noted that density of health workforce in India is less than half of the WHO recommended density threshold of 44.5 skilled health workers per 10 000 persons required for achieving the UHC and SDGs. Similar concerns have been raised about the low quality of health workforce both in the public and private sectors [20, 21]. The reported shortages are more severe for nurses/midwives compared to doctors leading to a highly inadequate skill-mix of doctors and nurses/midwives [12, 17, 22]. Improved absorption capacity (demand) of public sector for health workforce is often cited as one of the measures to improve health workforce availability for the population [12, 23, 24]. Most of the previous studies recommended the need for increased production of health professionals, also reflecting a large difference between the registration/stock of health professionals and active health workforce in India [7, 12, 18, 19]. The reported difference is to the extent of 40–50% with the latter being lower.

There are several reasons why qualified health professionals are not part of the current active health workforce. Past studies noted that the registration council’s data are not regularly updated to account for attrition of qualified health professionals due to migration, death and retirement. Also, there are issues with double counting in the registration data. Moreover, a recent study reflects that more than 30% of doctors and more than 50% of nurses with adequate qualifications are not part of the current health workforce just because they do not wish to join the labour market. In addition, there are also adequately qualified health professionals who are unemployed (4%) and working in non-health sectors [12].

India has taken many initiatives to overcome the HRH-related challenges. Recently the government of India constituted National Medical Commission (NMC) by an Act of Parliament with the aim and objectives of improving equity and quality in medical education on the one hand and encouraging the use of latest technologies, following ethical values and community perspective on the other [25]. The government also started centrally sponsored scheme to upgrade district hospitals to medical colleges and build new medical colleges with fund allocation in the range of INR 1 890 million to INR 3 250 million [26]. While, for increasing the intake capacity of MBBS seats in existing medical colleges, 12 million per seat is being allocated for strengthening and up-gradation of medical colleges in states [27]. Similarly, the state of Gujarat allocated 60% share of INR 600 million to seat upgradation from 150 to 250 seats in medical colleges [28]. In the 11th five-year plan (2007–12), INR 60 million was allocated to states, for upgrading a school of nursing attached to a medical college into a college of nursing [29, 30]. Also, INR 265 million is allocated for the construction of nursing colleges in the state of Bihar [31]. *NITI Aayog’s* strategy for “New India@75” also aims at creating 1.5 million jobs in the public health sector, creating more employment opportunities, primarily for women by 2022–2023 [32]. Similarly, *NITI Aayog’s* annual report 2019–20, re-emphasizes the importance of nursing sector reforms, providing quality education and structural reforms for maximizing their productivity [33].

Although past studies have documented various concerns related to HRH in India, the strategies and the magnitude of required investments to overcome these challenges have not been adequately discussed. The main objectives of the present study are threefold: (i) estimate the magnitude of HRH (doctors and nurses/midwives) shortages currently and for the year 2030; (ii) estimate quantum of investment required for production of additional health workforce to bridge the gaps by 2030; and iii) estimate potential benefits of such investments in terms of employment generation in health sector and contribution to national income.

## Materials and methods

The present study uses data from a range of sources: (i) National Health Workforce Accounts (NHWA) [34]; (ii) Periodic Labour Force Survey (PLFS), July 2018- June 2019, National Sample Survey Office (NSSO), Government of India, [35]; (iii) annual supply of new graduates (doctors and nurses/midwives) from the National Medical Commission (NMC) and Indian Nursing Council (INC) [36, 37]; (iv) population projection from Census of India 2019 [38]; and (v) websites of different government and private medical/nursing educational institutions. In addition to this, we also undertook a detailed review of literature from government and private sources providing information on unit costs of opening a new institution/seat expansion for doctors and nurses/midwives.

### NHWA data

NHWA provides country-wise data on the stock of different categories of health workers. The latest data on HRH stock for India are available for 2018. The present study uses all-India level data of two health professional categories: doctors and nurses/midwives.

### NSSO data

The sample size of the PLFS, July 2018–June 2019 is 101 579 households (55 812 rural and 45 767 urban) and 420 757 persons (239 817 rural and 180 940 urban). The survey provides information on detailed activity status, employment status, sector of employment and occupation types of each worker, educational achievements of each individual along with other socio-economic and demographic backgrounds of each individual covered in the sample [35].

### Estimation of total health workforce

We estimated the size of health workforce in terms of total production, total stock and active health workforce. The ‘total production’ represents the total number of doctors registered with NMC and nurses/midwives registered with INC and collated in the NHWA database. We defined ‘total stock’ after accounting for net migration (outmigration – in-migration), deaths and retirements of health professionals from the total produced health professionals. Using PLFS 2018–19, we estimated ‘active health workforce’ as the health professionals actually working in human health services [12]. The PLFS 2018–2019 could not identify disaggregated numbers of health professionals by allopathic doctors, AYUSH doctors and dentists employed in hospital settings. We applied the ratio of different health professionals outside the hospital sector to the hospital sector to arrive at the total estimate of different categories of health workers [12].

Using different data as explained above, we estimated the baseline number of doctors and nurses/midwives as of January 2021 and projected the estimates for each year between 2021 and 2030. For projection of total production and stock of health professionals and active health workforce, we used standard methods as discussed in Ridoutt et al. [39]. However, we used a range of indicators from India to modify the method for the present analysis purpose (Eqs. 1 and 2). See also the Supplementary document for details.

1 $${S}_{ht}={P}_{ht}-\left({M}_{ht}+{D}_{ht}+{R}_{ht}\right),$$

and

1a $${P}_{ht}={P}_{h2020}+ {s}_{ht-5},$$

where ‘*S*’ is total stock of health professionals and subscripts ‘*h*’ and ‘*t*’ ate types of health professionals and year of estimation, respectively. *M, D* and *R* are total net migration, deaths and retirement of health professionals. ‘*s*’ is total annual admission of medical students across all institutions in India.

2 $${HW}_{ht} = \,({S}_{h2020}-\left({OW}_{2019}+{U}_{2019}+{NW}_{2019}\right))+ {(s}_{ht-5}-({OW}_{ht}+{U}_{ht}+{NW}_{ht})),$$

where *HW* is active health workforce, *NW* is health professionals out-of-labour force, *U* is unemployed, and *OW* is health professionals working in non-human health services. *OW*, *U* and *NW* were estimated from PLFS 2018–19 and were used for the future years up to 2030. We marginally moderated (downward) these rates for the future years with the assumption that these rates may decline in future with the growing demand for health workers.

The shortage of HRH was estimated as the difference between the estimated density of HRH per 10 000 persons and the ILO & WHO recommended different density thresholds. The required levels of investment to bridge the gaps by the year 2030 were estimated using alternative assumptions of increasing the number of seats in the existing institutions and opening new institutions (Table 1) and the unit cost of increasing one seat and opening a new institution respectively. The proposed different scenarios represent different bounds of investments: (1) lower bound, to overcome the projected shortages in total stock; (2) upper bound, to overcome the projected shortages in active health workforce; and (3) middle bound, by reducing at least 50% of the existing labour market attrition by 2030. Two alternative scenarios are also presented: (i) if all proposed seat expansion is considered only in government institutions and (ii) if indigenous medicine (*Ayurveda, Yoga, Unani, Siddha and Homeopathy* [AYUSH]) practitioners are considered as a part of total health workforce.

**Table 1:** Detailed strategy for increased production and to overcome HRH shortages by 2030

Strategy	Strategy details	Doctors: Strategy at different thresholds	Nurses/midwives: Strategy at different thresholds	Investment bound
Strategy 1	To overcome projected shortages in actual stock	**34.5 & 44.5: **Seat expansion + Opening new colleges	**34.5:** Full utilization of existing capacities + Seat expansion (no new colleges required) **44.5:** Full utilization of existing capacities + Seat expansion + Opening new colleges	Lower bound of investment
Strategy 2	To overcome projected shortages in active health workforce	**34.5 & 44.5:**Seat expansion + Opening new colleges	**34.5 & 44.5:** Full utilization of existing capacities + Seat expansion + Opening new colleges	Upper bound of investment
Strategy 3	To overcome projected shortages in active health workforce by reducing at least 50% of the existing labour market attrition by 2030	**34.5 & 44.5:**Seat expansion + Opening new colleges + Encouraging and reskilling 50% of out-of-labour health professionals to join workforce	**34.5 & 44.5:** Full utilization of existing capacities + Seat expansion + Opening new colleges + Encouraging and reskilling 50% of out-of-labour health professionals to join workforce	Middle bound of investment
Strategy 4	Scenario 1: Considering seat expansion in only government institutions *- Lower bound of investment* *- Middle bound of investment* *- Upper bound of investment*	**34.5 & 44.5**: Seat expansion (only in government colleges)+ Opening new colleges (In addition, middle bound of investment includes encouraging and reskilling 50% of out-of-labour health professionals to join workforce)	NA	Investment range using alternative strategies and scenarios for doctors
	Scenario 2: Considering indigenous medicine (AYUSH) practitioners as part of health workforce	**34.5**: Seat expansion + including AYUSH practitioners (no new colleges required) **44.5:** Seat expansion + opening new colleges + including AYUSH practitioners	NA	

Numbers in bold are the two reference thresholds of *HRH* population ratio. NA is not applicable

Finally, we estimated the projected return to investment for the year 2030 by using employment data of associate health professionals and support staff, Gross Value Added (GVA) data and GVA per worker in health sector (Eqs. 3 and 4):

3 $${\Delta E}_{h2030}={\Delta HW}_{h2030}+{\Delta SS}_{h2030}+{\Delta HA}_{h2030},$$

where ${\Delta E}_{h2030}$ is additional number of all workers employed in health sector by the year 2030, ${\Delta HW}_{h2030}, {\Delta SS}_{h2030}\, and {\Delta HA}_{h2030}$ are additional health workforce (doctors and nurses/midwives), support staff and health associates, respectively, in the year 2030 due to the enhanced investment.Finally, contribution to national incomes was estimated as an increase in gross value added by the increased employment as follows:

4 $${\Delta GVA}_{h2030}=\Delta {E}_{h2030}*{LP}_{h2019},$$

and

4a $${{LP}_{h2019}=GVA}_{h2019}/{E}_{h2019,}$$

where ${\Delta GVA}_{h2030}$ is changes in gross value added in health sector and ${LP}_{h2019}$ is labour productivity of health sector workers and ${GVA}_{h2019}$ is gross value added in health sector in 2019 at current prices.

The detailed estimation procedures are presented in an Additional file 2.

## Results

### Current size and density of HRH

Using NSSO and NHWA data, we estimated the current size of HRH at the all-India level (Table 2). Mainly, three parameters are presented: (1) total production of health professionals; (2) actual stock of health professionals; and (3) active health workforce. NHWA data reported 1.16 million doctors and 2.34 million nurses/midwives as total production in the country as of 2018. The data also record approximately 0.79 million AYUSH practitioners.

**Table 2:** Size and composition of HRH in India

Parameters	Total production, 2018		Actual stock, 2018*		Active health workforce 2019^		Active health
	Number (in million)	Density/10 000 population	Number (in million)	Density/10 000 population	Number (in million)	Density/10 000 population	Workforce as % of actual stock
Allopathic doctor	1.16	8.8	1.05	7.9	0.66	5	63
Nurses/midwives	2.34	17.7	2.18	16.5	0.79	6	36
Allopathic doctors + nurses/midwives	3.5	26.5	3.23	24.4	1.45	11	45
AYUSH practitioners	0.79	6	0.76	5.8	0.25	1.9	33
Allopathic doctors + nurses/midwives + AYUSH	4.29	32.5	3.99	30.2	1.7	12.9	42.6

Sources: NHWA 2018 and PLFS 2018–19

*Adjusted for attrition (Out-migration): Allopathic Doctors: (− 6%); Nurses: (− 3.3%), death rate (for both nurses and doctors:( − 2.5% to − 2.1%), retirement rate(doctors):(− 1.07%), retirement rate(nurses):(− 1.02%); ^Estimated from PLFS:2018–2019 after accounting for adequate qualifications and population projection as of January 2019 (MoHFW 2019)

Total stock of health professionals as of 2018 is estimated to be 1.05 million doctors and 2.18 million nurses/midwives. However, the size of the estimated active health workforce, is considerably lower, with 0.66 million doctors and 0.79 million nurses/midwives. At the aggregate level, adding numbers of doctors and nurses/midwives together the size of active health workforce is around 45% of the total actual stock of health professionals. Accordingly, the density, of health professionals available in stock is 24.4 per 10 000 population when considering only allopathic doctors and nurses/midwives. However, including AYUSH professionals the density of health worker stock increases to 30.2 per 10 000 population. The density of active health workforce is estimated to be around 11 and 12.9 by excluding and including AYUSH, respectively.

As far as skill-mix of HRH is concerned, doctor:nurse/midwives ratio is estimated to be 1:2 in the production and stock data, while active health workforce data reflect 1:1.2 ratio. This essentially reflects that proportion of qualified nurses/midwives not active in human health service is much larger as compared with that of doctors [12, 18].

### Supply-side estimates of HRH

Table 3 presents the projections of total cumulative production, stock of health professionals and active health workforce, separately for doctors, nurses/midwives and AYUSH practitioners by the year 2030. As of 2030, total cumulative production will be 2.06 million doctors while only a little over half of this supply (1.1 million) will be working in health services. While the actual available stock of nurses/midwives will be 2.74 million by 2030, only about half (1.4 million) of this stock will be active health workforce.

**Table 3:** Projected estimates of size and density of HRH, by 2030

Parameters	Doctors	Nurses/midwives	Doctors + nurses/midwives	AYUSH	Doctors + nurses/midwives + AYUSH
Total production of health professionals (in million)*	2.06 (14.1)	3.94 (26.9)	6 (41)	1.29 (8.8)	7.29 (49.8)
Total stock of health professionals (in million)**	1.51 (10.3)	2.74 (18.7)	4.25 (29)	0.93 (6.4)	5.18 (35.4)
Active health workforce (in million)^	1.1 (7.5)	1.41 (9.6)	2.51 (17.1)	0.51 (3.5)	3.02 (20.6)

Sources: NHWA 2018; PLFS 2018–19 and MoHFW 2019

*includes estimated pass-outs from all institutions established and announced to be established by 2025; **adjusted for attrition (mortality, retirement and migration); ^Estimated from PLFS:2018–19, moderate labour market attrition of 20%-doctors and 30%-Nurses and attrition (mortality, retirement and migration). Figures in parentheses are density per 10 000 persons. Doctors-Net migration rate:(+ 5%), death rate:(− 2.5 to − 2.1%), retirement rate:(− 1.07%) and Nurses(/midwives)-Annual Migration:(− 4.6%), death rate:(− 2.5 to − 2.1%), retirement rate:(-1.02%)

Moreover, the total stock of HRH including doctors and nurses/midwives by the year 2030 will be about 4.25 million and 5.18 million without and with AYUSH professionals, respectively. However, there will be only 2.51 million doctors and nurses/midwives in the active health workforce. Including AYUSH in the workforce, the number of active health workers increases to 3.02 million by 2030. The density of health professionals is about 29 per 10 000 persons when considering the stock, which comes down to 17.1 skilled doctors and nurses/midwives in the active health workforce. If we include AYUSH professionals, the density is around 20.6 skilled doctors and nurses/midwives in the active health workforce.

### Health worker shortages at different thresholds

**Fig. 1:**
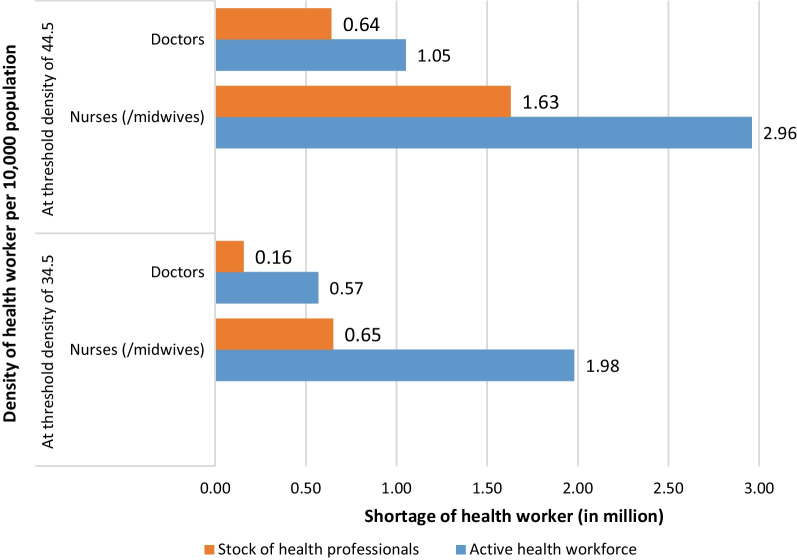
Estimates of shortages (in million) of health worker by 2030 at different health workers–population density thresholds per 10 000 population. Sources: NHWA 2018; PLFS 2018-2019 and Census of India 2011. Note: With an assumed, doctors:nurses/midwives ratio of 1:2

The required number of doctors and nurses/midwives to meet the overall HRH:population ratio thresholds of 34.5 and 44.5 per 10 000 population [2, 4, 40–44] were estimated assuming a doctor:nurses/midwives ratio of 1:2 (Additional file 1: Table S1). The stock shortage for doctors is 0.16 million by the year 2030 at the 34.5 density threshold (Fig. 1). The shortages at the same threshold are much higher (0.57 million) for doctors in the active health workforce. At the density of 44.5, both the stock and active health workforce are reporting doctor shortages of 0.64 million and 1.05 million, respectively. The nurse shortage in stock reaches up to 0.65 million by the year 2030 to meet the density threshold of 34.5. The shortages at the same threshold are more than three-folds (1.98 million) if we consider the number of nurses/midwives actively working. At the density threshold of 44.5 skilled health workers per 10 000 population, the shortages of nurses/midwives in the stock and active health workforce are estimated to be approximately 1.63 million and 2.96 million, respectively, by 2030.

### Strategies and required levels of investment

The strategies to increase the production of doctors and nurses/midwives by expanding the seat capacity of the existing institutions or opening new institutions or both are presented in Table 4. Total number of existing and upcoming institutions by 2025 are 675 medical colleges and 7 110 nursing institutes with a respective seat capacity of 95 325 and 289 000 (Additional file 1: Table S2). Given the total number of institutions and average annual pass-outs of 136 doctors and 19 nurses/midwives per institution, there is significant scope to increase the number of seats and institutions. However, for the increased production of nurses/midwives, improved utilization of the existing capacities would be crucial. Currently only 19 nurses on average per institution pass-out annually with the existing capacity of 41 seats per nursing institution.

**Table 4:** New investment required for meeting the doctors and nurses/midwives shortages at different health worker:population ratio thresholds, by 2030

Parameters	Health worker density threshold^	Required new production (‘000’) per annum during 2021–2025*	Required number of seat expansion per college (total seats ‘000’)	Required number of new colleges (total seats ‘000’)	Estimated total cost of investment (in INR billion)^^
*Lower bound of investment—actual stock shortages*					
Doctors	34.5	39	39 (26)	87 (13)	523
	44.5	160	39 (26)	892 (134)	2 941
Nurses/midwives	34.5	161	21 (149) + 2(14)**	0	0
	44.5	406	21 (149) + 20 (142)***	1 918 (115)	707
*Upper bound of investment—active health workforce shortages*					
Doctors	34.5	142	39 (26)	772 (116)	2 580
	44.5	263	39 (26)	1 578 (237)	4 998
Nurses/midwives	34.5	494	21 (149) + 20 (142)***	3 385 (203)	1 096
	44.5	740	21 (149) + 20 (142)***	7 475 (448)	2 180
*Middle bound of investment—by considering 50% of the out of workforce qualified health personnel into active health workforce^^^*					
Doctors	34.5	95	39 (26)	458 (69)	1 636
	44.5	216	39 (26)	1 263 (189)	4 053
Nurses/midwives	34.5	357	21 (149)*** + 20 (142)	1 100 (66)	491
	44.5	603	21 (149)*** + 20 (142)	5 190 (311)	1 574

^ Skilled health worker density per 10 000 population; *Required production per annum for a duration of 4 years (Total required production/4); ^^Doctors: The investment estimates includes cost of seats (INR 10 million per seat) expansion in existing (/proposed) colleges and cost of opening new institutions (INR 3 000 million per institution) and for nurses/midwives: The investment estimates includes cost of seats (INR 1.4 million per seat) expansion in existing (/proposed) colleges and cost of opening new institutions (INR 265 million per institution);**includes increasing pass-out rate in existing institution by 21 seats per institution (no cost involved) and seat expansion by 2 per institution (cost not considered for increasing 2 seat per institution);***includes increasing pass-out rate in existing institution by 21 seats per institution (no cost involved) and seat expansion by 20 per institution (INR 1.4 million per seat); ^^^Doctors: Annual shortages estimated after including 50% of medically qualified health professionals who are not part of health workforce (0.19 million doctors) to the total shortages by 2030 and in nurses/midwives, annual shortages estimated after including 50% of medically qualified health professionals who are not part of health workforce (0.55 million nurses/midwives to the total shortages by 2030

Given the levels of infrastructure in the existing institution, an increase of 35–40 seats per institution in medical colleges and 20 seats per institution in nursing institutes is possible. The remaining shortages can be bridged by opening new institutions. Increasing 35–40 seats in medical colleges and 20 seats in nursing institutions on average will lead to an average seat capacity of 170–175 seats per medical college and 61 seats per nursing institute (Table 4). In such a scenario an additional 87 medical colleges with similar seat capacity will be required to meet the stock shortage of doctors at the 34.5 density threshold. There will be no need of opening new nursing institutes as an improved pass-out rate will be almost equal to the stock shortages in nurses at the 34.5 density threshold. However, to bridge the stock shortages at the 44.5 thresholds, there will be a requirement of opening 892 new medical colleges and 1 918 new nursing institutions along with seat expansion in the existing institutions.

Further, to bridge the shortages of active health workforce, along with the seat expansion there will be a requirement of opening 772 new medical colleges and 3 385 new nursing institutes at the 34.5 threshold and 1 578 medical colleges and 7 475 nursing institutes at the density threshold of 44.5. With lower seat expansion, the shortages can be met only by opening a higher number of institutions ranging from 87 and 1 691 medical colleges depending on the gaps to be met in stock or active health workforce on the one hand and 34.5 and 44.5 density thresholds on the other (Additional file 1: Table S3).

Accordingly, the required levels of investment were estimated by applying unit costs (INR 10 million for one seat expansion and INR 3 000 million for opening one medical college for doctors and INR 1.4 million for one seat expansion and INR 265 million for opening one new nursing institute) over the total number of seat expansion and new institutions required to be increased. The size of the required investment varied depending on the gaps to be met in stock or active health workforce on the one hand and the density thresholds on the other. Meeting the HRH shortage and required investment in stock and active health workforce are two extreme bounds (call it lower bound and upper bound). To meet the stock gaps, the required investment ranged between INR 523 billion and INR 2 941 billion at the density thresholds of 34.5 and 44.5, respectively. For the stock of nurses, the required investment is about INR 707 billion at the density threshold of 44.5. However, bridging the shortages of active health workforce at the 34.5 density threshold, investment requirements are INR 2 580 billion for doctors and 1 096 for nurses/midwives. At the 44.5 threshold, the investment requirements are INR 4 998 billion for doctors and INR 2 180 billion for nurses/midwives.

We also present a medium bound of investment (Table 4), which has been estimated by considering inclusion of 50% of the qualified health professionals who are out of the labour force. If efforts are made to attract at least 50% of the out-of-labour force health professionals to be part of the active health workforce, there will be an investment requirement of INR 1 636 and INR 4 053 billion at the density thresholds of 34.5 and 44.5, respectively, for bridging the shortage of doctors by 2030. For nurses/midwives, the respective investment requirements are estimated to be INR 491 and INR 1 574 billion. Different other scenarios of combinations of seat expansion and opening new institutions are presented in Additional file 1: Table S4.

**Fig. 2:**
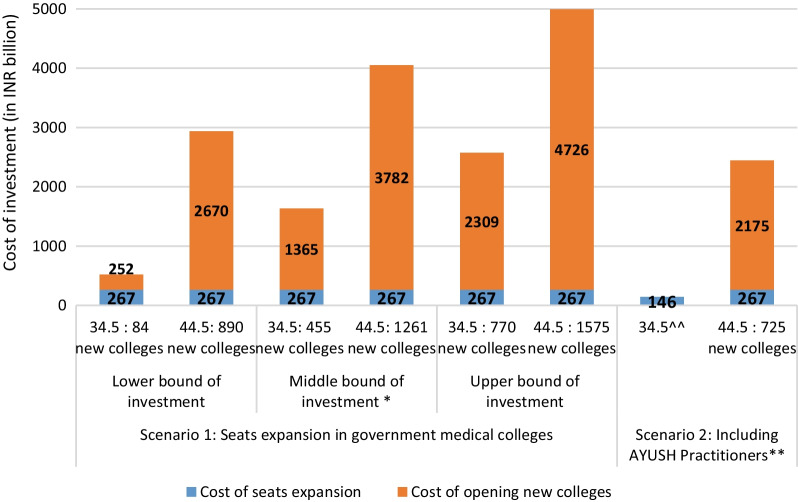
Strategy 4 and required investment (in billion) to overcome doctor’s shortages by 2030, using government colleges and including AYUSH practitioners^. Note: *Investment required to overcome annual shortages, estimated after adopting scenario 1 (Strategy 4)-Including 50% of medically qualified health professionals who are not part of health workforce (0.19 million doctors) to the total shortages by 2030; **Investment required to overcome annual shortages estimated including 0.51 AYUSH practitioners to the total shortages by 2030; ^Doctors: Required production per annum for a duration of 4 years (Total required production/4), investment estimates includes cost of seats expansion (INR 10 million per seat) in existing (/proposed) colleges and cost of opening new institutions (INR 3 000 million per institution); ^^with seat expansion by 22 seats per institution (no new colleges required)

In yet another alternative scenario, we only considered seat expansion in government medical colleges. In this scenario, we estimated an additional capacity of 26 726 seats in government medical colleges by increasing the seat intake up to 200 per college, exclusively in colleges with current uptake below 200. The required investment under this scenario is estimated to be INR 519 to 2 576 billion at the 34.5 threshold and INR 2 973 to 4 993 billion at the 44.5 threshold (Fig. 2). We also estimated the potential shortages in doctors and the related investment requirement by considering AYUSH as part of active health workforce (Fig. 2) and the estimated investment requirement for bridging the doctors’ shortage by 2030 is INR 146 and INR 2 446 billion at the threshold 34.5 and 44.5, respectively (Additional file 1: Table S4).

### Economic benefits of investment in HRH, by 2030

Although investment in HRH has multiple pathways to economic growth [8, 45], in the present study we only estimated potential benefits in terms of employment generation and labour productivity. We used the quantum of investment required for overcoming the HRH shortages in active health workforce as the benchmark for estimating the potential employment generation and labour productivity benefits. At the density threshold of 34.5, the estimated upper bound of investment of INR 3 676 billion has the potential to create employment for 5.4 million health workers. The additional employment of 2.55 million, consisting of doctors and nurses/midwives can generate new 1.51 million employment of junior support staff and 1.35 million health associate personnel separately, after adjusting for a labour market attrition rate of 20%. The labour productivity (GVA/employment) per worker in the health sector for the year 2019–2020 is estimated to be INR 633 thousand. Using this estimated labour productivity for 2030 reflects that the marginal GVA (due to the additional employment generated by 2030) could be INR 3 429 billion during 2026–2030 (Table 5).

**Table 5:** Estimates of benefits of investment in terms of new employment and contribution to national gross value added by 2030

Parameter	Required HRH investment during 2021–25 (INR billion)	New employment generation (in million)				Total gross value added during 2026–2030 (INR billion)
		Health workers	Support staff*	Health associate**	Total	
34.5 skilled health worker per 10 000 population	3 676	2.55	1.51	1.35	5.42	3 429
44.5 skilled health worker per 10,000 population	7 178	4.01	2.38	2.13	8.52	5 392

*Supportive staff include other support workers such as administrators, clerks, accountants, motor drivers, garbage collectors, etc.; **Includes health associates such as nutritionists, dieticians, optometrists, etc.

At the higher density threshold of 44.5, the benefit in the health sector is to the tune of INR 5 392 billion annually with the required investment of INR 7 178 billion. Here, it is important to note that the estimated investment is a one-time requirement to be completed between 2021 and 2025 but the contribution to GVA because of the increased employment will be each year for a long period of time. If the required investment is spread over a period of 4 years (during 2021–25), the same is estimated to be 0.4% and 0.8% of GDP at the 34.5 and 44.5 density thresholds, respectively.

## Discussion and policy implications

The returns of investment in HRH are emphatically emphasized in literature as its effects are many folds, including beyond the health sector [2, 3, 8, 45]. In the Indian context, it not only complements the initiatives taken towards achieving UHC, but also has the potential to generate employment, improve female labour force participation and increase the formalization of labour market if commensurate measures are undertaken to increase the demand for health workers in health system. Past studies have highlighted acute shortage and skewed distribution of health workforce in India and the need to invest in improving the HRH:population ratios are the need of the hour [10, 12, 15, 18].

The present study estimated the health worker shortages and the required magnitude of investment to overcome the shortages and meet the ILO and WHO recommended thresholds of HRH:population ratio with a supply-side perspective. Past studies highlighted the need for multipronged strategies, to bridge the shortages in health workforce, which range from increased production of doctors and nurses/midwives [23] to raising the demand for health workers at the health system level and filling up any existing vacancies at the facility levels [12, 18]. Also, improving the working conditions and remuneration of doctors and nurses/midwives has the potential to increase the number of graduates that enter the health workforce [46, 47]. Since many of these strategies may not involve a substantial new investment and can be addressed with annual increased budgetary allocation to health sector, we focused on estimating the required capital investment for increased production of health workforce.

When considering the active health workforce shortages, the required investment is much higher to meet the shortages compared to the shortages in the stock of health professionals registered with medical and nursing councils. The investment amount ranges from INR 523 billion to INR 2 580 billion for doctors and 1 096 billion for nurses, respectively, to meet 34.5 HRH: population threshold. However, the required investment is much higher INR 3 thousand to 7 thousand billion to meet the density threshold of 44.5.

One of the main reasons for the differences in actual stock and active health workforce is labour market attrition, around 30% of health professionals with a degree in medicine are not in the labour force and only 40% of health professionals with a diploma in medicine are the part of the current health workforce [12]. If efforts are made to engage 50% of these not-working (out-of-labour force) professionals into the active health workforce, by providing an improved work environment, flexible working hours, raising the retirement age limit, etc., the shortages in health workforce significantly declines and the required investment to bridge the gap in active health workforce comes down by 20%. Further, by including both AYUSH practitioners and engaging 50% out-of-labour workforce in active health workforce, there will be no shortages of doctors to meet 34.5 HRH:population thresholds. However, the required investment will still be approximately INR 2.5 thousand billion to meet the threshold of 44.5.

It is important to attract talent to join the nursing profession by incentivizing nursing education and by providing attractive employment opportunities. Improvising the quality of nursing education and achieving higher pass-out rates from existing institutions can improvise the nurse’s availability in the workforce. Creating a national licensing exam for nurses/midwives could be considered an instrument to improve the quality of education. Factors such as low demand for trained nurses, poor quality of nursing education, lack of non-technical skills and knowledge, desire to opt for higher education and a range of socio-economic factors are some of the reasons affecting the employment opportunities of nurses [48]. Also, a low retention rate of nurses in the system has been one of the most important reasons for the shortage of nurses in the active workforce [49]. There are only a few studies available to understand the nurse turnover rates in India. Past studies reported about 28–35% attrition rates among nurses, as a large proportion of them are either not absorbed in the health system or leave employment after a few years of working. Poor working conditions and low remuneration are often cited as the reasons [46, 50]. Out-migration of health professionals is also a challenge, a major proportion of the workforce, 6.6% and 3% of doctors and nurses, respectively, registered within Indian councils are working in OECD countries [51]. Thus, it is important to recognize these challenges and concerns, to have an adequate skill-mix balance. More detailed and in-depth studies are required to understand the reasons for low turnover and lack of employment opportunities for nurses and midwives in India.

On the supply side, the distribution of institutions is lopsided in the country, where public institutions produce only half of the total number of new graduate doctors, while in the case of nurses, the majority (90%) of educational institutions are in the private sector [36, 37]. The estimated volume of investment can be distributed to balance the situation between the public and private sectors. Distributing the opening of the proposed new institutions in less developed states and closer to remote areas usually demonstrate greater retention of doctors in local areas. However, there is a need to take care of the quality of educational institutions in remote areas [52].

Moreover, the strategies should be not only the opening of new institutions and expanding intake capacity. Greater emphasis to increase current public health spending and attracting talent to join the nursing workforce, along with preparing and investing in healthcare settings to absorb them, are the required supporting measures [9, 23].

The estimated marginal gross value addition because of increased employment of 5.42 million new workers is to the tune of annual INR 3 429 billion at constant 2019–2020 prices. However, once the shortages are met with these one-time investments during the period 2021–2025, the returns will be perpetual. For instance, if the estimated investment is made during the next 1–2 years, the return on the investment will be 5 times higher than the estimated annual marginal value added of INR 3 429 (for the period 2026–2030) as of 2030. Thus, these investments not only help towards achieving UHC and SDGs but will also contribute to the national income.

### Limitations

The study only estimated the one-time capital investment needed for producing increased numbers of doctors and nurses. The study did not estimate the required annual recurring expenditure likely to be incurred on faculty recruitment, consumables, maintenance, etc., on the supply side and raising the demand for health workers, improving working conditions and remuneration, etc., on the demand side. Also, the estimated required investment is likely to be underestimated, if the distribution of new institutions is required to be opened in remote areas, on account of addressing quality issues. Similarly, the size of the benefits estimated in this study is only limited to potential employment generation and labour productivity in the health sector and only for one year. However, the benefits of such investments are expected to flow beyond the health sector for many years. The study recommends that a more detailed study for estimating the total costs of investments and benefits should be conducted.

## Conclusions

India needs to significantly increase the level of investments in the production of health workforce and mainstream them into an active health workforce. Although several interventions such as improving working conditions of health workers, raising the demand for health workers in the health system, filling up existing vacancies and a continuum of training and skill development are required to bridge the existing and potential shortage of health workers, new investments in the production of health workforce have the potential to benefit India within and beyond health sector.

## Supplementary Information

**Additional file 1. Table S1. **Required annual and total production of doctors and nurses/midwives for overcoming stock HRH shortages, by 2030. **Table S2. **Supply side scenario of medical colleges and nursing institution in India. **Table S3. **Strategies for production of doctors and nurses/midwives to overcome HRH shortages, by 2030. **Table S4.** Alternative scenarios for overcoming doctors and nurses/midwives shortages at different health worker thresholds.

**Additional file 2.** Estimation of Investment Need for Increased Production of Health Workforce.

## Data Availability

Data for this study were used from secondary sources. Micro data from the NSSO are available for free in public domain from the official website (http://microdata.gov.in/nada43/index.php/catalog/146) of the National Sample Survey Office, Ministry of Statistics and Programme Implementation, Government of India.

## Abbreviations

ANM: Auxiliary Nurse Midwife
AYUSH: Ayurveda, Yoga and Naturopathy, Unani, Siddha and Homeopathy
BRICS: Brazil, Russia, India, China and South Africa
ComHEEG: High-Level Commission on Health Employment and Economic Growth
CSS: Centrally sponsored scheme
GNM: General Nurse Midwife
GVA: Gross value added
HRH: Human resources for health
ILO: International Labour Organization
INR: Indian National Rupee
LMICs: Lower and Middle Income Countries
MoHFW: Ministry of Health and Family Welfare
NHP: National Health Policy
NHWA: National Health Workforce Account
NITI Aayog: National Institution for Transforming India
NSSO: National Sample Survey Office
OECD: Organization for Economic Cooperation and Development
PLFS: Periodic Labour Force Survey
SDG’s: Sustainable Development Goals
UHC: Universal health coverage
UN: United Nations
WHO: World Health Organization
WPR: Worker population ratio
