# Supplementary material for: Achieving universal health coverage and sustainable development goals by 2030: investment estimates to increase production of health professionals in India
Source: Hum Resour Health. 2023 Mar 2;21:17. doi: 10.1186/s12960-023-00802-y (PMC9979880; doi:10.1186/s12960-023-00802-y)
Supplement: Supplementary file 1 — Additional file 1. Table S1. Required annual and total production of doctors and nurses/midwives for overcoming stock HRH shortages, by 2030. Table S2. Supply side scenario of medical colleges and nursing institution in India. Table S3. Strategies for production of doctors and nurses/midwives to overcome HRH shortages, by 2030. Table S4. Alternative scenarios for overcoming doctors and nurses/midwives shortages at different health worker thresholds. [file 12960_2023_802_MOESM1_ESM.docx]

**Additional file 1: tables**

Additional file 1: Table S1. Required annual and total production of doctors and nurses/midwives for overcoming stock HRH shortages, by 2030

| Estimated shortages of doctors and nurses/midwives in stock  and active health workforce, by 2030 | | |
| --- | --- | --- |
| **Actual stock shortages** | | |
| Total required production, by 2030 | | |
| **Recommended threshold** | **34.5** | **44.5** |
| Nurses/midwives (In million) | 0.65 | 1.63 |
| Doctors (In million) | 0.16 | 0.64 |
| Required annual production with duration of course- 4 years (Required production / 4) | | |
| Nurses/midwives (In million) | 0.16 | 0.41 |
| Doctors (In million) | 0.04 | 0.16 |
| **Active workforce shortages** | | |
| Total required production, by 2030 |  |  |
| **Recommended threshold** | **34.5** | **44.5** |
| Doctors (In million) | 0.57 | 1.05 |
| Nurses/midwives (In million) | 1.98 | 2.96 |
| Required annual production with duration of course- 4 years (Required production / 4) | | |
| Doctors (In million) | 0.14 | 0.26 |
| Nurses/midwives (In million) | 0.49 | 0.74 |

Sources: NHWA 2018; PLFS 2018-19 and Census of India 2011.

Additional file 1: Table S2. Supply side scenario of medical colleges and nursing institution in India

|  | Medical colleges^ | | | Nursing institutions# | | |
| --- | --- | --- | --- | --- | --- | --- |
| Parameters | Government | Private | All | Government | Private | All |
| **Existing institutions up to 2020** | | | | | | |
| *Number of institutions* | 276 | 278 | 554 | 761 | 6,312 | 7,073 |
| *Number of seats* | 41,610 | 41,340 | 82,950 | 33,150 | 2,53,387 | 2,86,537 |
| *Seats per institution* | 151 | 149 | 150 | 44 | 40 | 41 |
| *Pass-outs per institutions** | 145 | 144 | 144 | 21 | 19 | 17 |
| **Institution announced to be set up between 2021-25** | | | | | | |
| *Number of institutions* | 121 | NA | 121 | NA | NA | 37 |
| *Number of seats* | 12,375 | NA | 12,375 | NA | NA | 2,220 |
| *Seats per institution* | 102 | NA | 102 | NA | NA | 60 |
| *Pass-outs per institutions** | 99 | NA | 99 | NA | NA | 28 |
| **Total number of institutions by 2025** | | | | | | |
| *Number of institutions* | 397 | 278 | 675 | NA | NA | 7,110 |
| *Number of seats* | 53,985 | 41,340 | 95,325 | NA | NA | 2,89,000 |
| *Seats per institution* | 136 | 149 | 141 | NA | NA | 41 |
| *Pass-outs per institutions** | 131 | 144 | 136 | NA | NA | 19 |

Note: NA is data not applicable; ^includes newly announced medical colleges and AIIMS. Assumption of availability of 100 seats/college in new medical colleges and 125 seats/new AIIMS; # Assumption of availability of 60 seats/new nursing colleges; *existing pass-out rate of 96.5% for doctors and new registrations/ training capacity of about 53% in ANM and 46% in GNM and BSc nurses based on latest 3 years data.

Additional file 1: Table S3. Strategies for production of doctors and nurses/midwives to overcome HRH shortages, by 2030

| Strategy | Investment bound | Strategy details | Doctors*: Strategy and scenarios at different thresholds^ | Nurses(/midwives)**: Strategy and scenarios at different thresholds^ |
| --- | --- | --- | --- | --- |
| Strategy 1 | Lower bound of investment | To overcome projected shortages in actual stock | Scenario I  **34.5 & 44.5:**  Seat expansion +  Opening new colleges | Scenario I  **34.5:** Full utilization of existing capacities +  Seat expansion (no new colleges required)  **44.5:** Full utilization of existing capacities +  Seat expansion + Opening new colleges |
|  |  |  | Scenario II  **34.5 & 44.5:**  Seat expansion +  Opening new colleges | Scenario II  **34.5:** Full utilization of existing capacities +  Seat expansion (no new colleges required)  **44.5:** Full utilization of existing capacities +  Seat expansion + Opening new colleges |
|  |  |  | Scenario III  **34.5 & 44.5:**  Seat expansion +  Opening new colleges | Scenario III  **34.5:** Full utilization of existing capacities +  Opening new colleges  **44.5:** Full utilization of existing capacities +  Opening new colleges |
| Strategy 2 | Upper bound of investment | To overcome projected shortages in active health workforce | Scenario I  **34.5 & 44.5:**  Seat expansion +  Opening new colleges | Scenario I  **34.5 & 44.5:** Full utilization of existing capacities +  Seat expansion + Opening new colleges |
|  |  |  | Scenario II  **34.5 & 44.5:**  Seat expansion +  Opening new colleges | Scenario II  **34.5 & 44.5:** Full utilization of existing capacities +  Seat expansion + Opening new colleges |
|  |  |  | Scenario III  **34.5 & 44.5:**  Seat expansion +  Opening new colleges | Scenario III  **34.5 & 44.5:** Full utilization of existing capacities +  Seat expansion + Opening new colleges |
| Strategy 3 | Middle bound of investment | To overcome projected shortages in active health workforce by reducing at least 50% of the existing labour market attrition by 2030 | Scenario I  **34.5 & 44.5:**  Seat expansion +  Opening new colleges +  Encouraging and reskilling 50% of out of labour health professionals to join workforce | Scenario I  **34.5 & 44.5:** Full utilization of existing capacities +  Seat expansion + Opening new colleges + Encouraging and reskilling 50% of out of labour health professionals to join workforce |
|  |  |  | Scenario II  **34.5 & 44.5:**  Seat expansion +  Opening new colleges +  Encouraging and reskilling 50% of out of labour health professionals to join workforce | Scenario II  **34.5 & 44.5:** Full utilization of existing capacities +  Seat expansion + Opening new colleges + Encouraging and reskilling 50% of out of labour health professionals to join workforce |
|  |  |  | Scenario III  **34.5 & 44.5:**  Seat expansion +  Opening new colleges +  Encouraging and reskilling 50% of out of labour health professionals to join workforce | Scenario III  **34.5 & 44.5:** Full utilization of existing capacities +  Opening new colleges + Encouraging and reskilling 50% of out of labour health professionals to join workforce |
| Strategy 4 | Investment scenarios for doctors | Scenario 1: Considering seat expansion in only government institutions  *-Lower bound of investment*  *-Middle bound of investment*  *-Upper bound of investment* | *-Lower bound of investment*  **34.5 & 44.5**: Seat expansion (only in government colleges)  + Opening new colleges  *-Middle bound of investment*  **34.5 & 44.5**: Seat expansion (only in government colleges)  + Opening new colleges + Encouraging and reskilling 50% of out of labour health professionals to join workforce  *-Upper bound of investment*  **34.5 & 44.5**: Seat expansion (only in government colleges)  + Opening new colleges | NA |
|  |  | Scenario 2: Considering indigenous medicine (AYUSH) practitioners as part of health workforce | Scenario I  **34.5**: Seat expansion + including AYUSH practitioners (no new colleges required)  **44.5:** Seat expansion  + Opening new colleges + including AYUSH practitioners | NA |
|  |  |  | Scenario II  **34.5**: Seat expansion + including AYUSH practitioners (no new colleges required)  **44.5:** Seat expansion  + Opening new colleges + including AYUSH practitioners | NA |
|  |  |  | Scenario III  **34.5**: Seat expansion  + Opening new colleges + including AYUSH practitioners  **44.5:** Seat expansion  + Opening new colleges + including AYUSH practitioners | NA |

Note: ^Skilled health worker density per 10,000 population;*For doctors: opening of new colleges (150 seats/college), scenario I involves expanding current average 136 seats to 175 seats in existing medical colleges, scenario II involves expanding current average 136 seats to 170 seats in existing medical colleges & scenario III involves expanding current average 136 seats to 150 seats in existing medical colleges;** For nurses: Utilizing capacities in existing nursing institutions to increase average pass out up to 40 pass-out/nursing college and opening new nursing colleges (60 seats/college), scenario I involves expanding current average 40 seats to 60 seats in existing nursing institutions, scenario II involves expanding current average 40 seats to 50 seats in existing nursing institutions and scenario III involves no seat expansion. NA is not applicable.

Additional file 1: Table S4. Alternative scenarios for overcoming doctors and nurses/midwives shortages at different health worker thresholds

| Parameters | Recommended health worker density threshold^ | Required new production (‘000’) per annum during 2021-25* | Required number of seat expansion per college (Total seats ‘000’) | Required number of new colleges (Total seats ‘000’) | Estimated total cost of investment (in INR billion)^^ |
| --- | --- | --- | --- | --- | --- |
| **STRATEGY 1: Lower bound of investment – Actual stock shortages** | | | | | |
| Doctors | | | | | |
| Scenario II | 34.5 | **39** | 34(23) | 96(16) | 518 |
|  | 44.5 | **160** | 34(23) | 915(137) | 2,974 |
| Scenario III | 34.5 | **39** | 14(9) | 199(30) | 692 |
|  | 44.5 | **160** | 14(9) | 1,005 (151) | 3,109 |
| Nurses(/midwives) | | | | | |
| Scenario II | 34.5 | **161** | 21(149) + 2(14)** | 0 | 0 |
|  | 44.5 | **406** | 21(149) + 10(71)*** | 3,103(186) | 922 |
| Scenario III | 34.5 | **161** | 21(149)*** | 198(12) | 53 |
|  | 44.5 | **406** | 21(149)*** | 4,288(257) | 1,136 |
| **STRATEGY 2: Upper bound of investment – Active health workforce shortages** | | | | | |
| Doctors | | | | | |
| Scenario II | 34.5 | **142** | 34(23) | 795(119) | 2,614 |
|  | 44.5 | **263** | 34(23) | 1,601(240) | 5,031 |
| Scenario III | 34.5 | **142** | 14(9) | 885(133) | 2,749 |
|  | 44.5 | **263** | 14(9) | 1,691(254) | 5,166 |
| Nurses(/midwives) | | | | | |
| Scenario II | 34.5 | **494** | 21(149) + 10(71)*** | 4,570(274) | 1,310 |
|  | 44.5 | **740** | 21(149) + 10(71)*** | 8,660(520) | 2,394 |
| Scenario III | 34.5 | **494** | 21(149)*** | 5,755(345) | 1,525 |
|  | 44.5 | **740** | 21(149)*** | 9,845(591) | 2,609 |
| **STRATEGY 3: Middle bound of investment- Active health workforce shortages**  (Active health workforce shortages by reducing at least 50% of the existing labour market attrition by 2030) # | | | | | |
| Doctors | | | | | |
| Scenario II | 34.5 | **95** | 34(23) | 480(72) | 1,670 |
|  | 44.5 | **216** | 34(23) | 1,286(193) | 4,087 |
| Scenario III | 34.5 | **95** | 14(9) | 570(86) | 1,805 |
|  | 44.5 | **216** | 14(9) | 1,376(206) | 4,222 |
| Nurses(/midwives) | | | | | |
| Scenario II | 34.5 | **357** | 21(149) + 10 (71)*** | 2,285(137) | 705 |
|  | 44.5 | **603** | 21(149) + 10 (71)*** | 6,375(382) | 1,789 |
| Scenario III | 34.5 | **357** | 21(149)*** | 3,470 (208) | 919 |
|  | 44.5 | **603** | 21(149)*** | 7,560(453) | 2,003 |
| **STRATEGY 4: Investment using alternative scenarios for doctors** | | | | | |
| Scenario 1: Considering seat expansion in only government institutions | | | | | |
| Lower bound of investment | 34.5 | **39** | 26 | 84(13) | 519 |
|  | 44.5 | **160** | 26 | 890(133) | 2,937 |
| Middle bound of investment | 34.5 | **95** | 26 | 455(68) | 1,632 |
|  | 44.5 | **216** | 26 | 1,261(189) | 4,049 |
| Upper bound of investment | 34.5 | **142** | 26 | 770(115) | 2,576 |
|  | 44.5 | **263** | 26 | 1,575(236) | 4,993 |
| Scenario 2: Considering indigenous medicine (AYUSH) practitioners as part of health workforce## | | | | | |
| Scenario I | 34.5 | **15** | 22(15)^^^ | 0 | 146 |
|  | 44.5 | **135** | 39(26) | 728(109) | 2,446 |
| Scenario II | 34.5 | **15** | 22(15)^^^ | 0 | 146 |
|  | 44.5 | **135** | 34(23) | 750(112) | 2,480 |
| Scenario III | 34.5 | **15** | 14(9) | 34(5) | 197 |
|  | 44.5 | **135** | 14(9) | 840(126) | 2,615 |

Note: ^Skilled health worker density per 10,000 population*Required production per annum for a duration of 4 years (Total required production/4);^^Doctors: The investment estimates includes cost of seats (INR 10 million per seat) expansion in existing (/proposed) colleges and cost of opening new institutions (INR 3,000 million per institution) and for nurses/midwives: The investment estimates includes cost of seats (INR 1.4 million per seat) expansion in existing (/proposed) colleges and cost of opening new institutions (INR 265 million per institution);**includes increasing pass-out rate in existing institution by 21 additional pass outs per institution (no cost involved) and seat expansion by 2 seats per institution (cost not considered for increasing 2 seats per institution);*** includes increasing pass-out rate in existing institution by 21 additional pass outs per institution (no cost involved) in both scenario II and III and seat expansion by 10 seats per institution in scenario II (INR 1.4 million per seat);# Doctors: Annual shortages estimated after Including 50% of medically qualified health professionals who are not part of health workforce (0.19 million doctors) and in nurses, annual shortages estimated after including 50% of medically qualified health professionals who are not part of health workforce (0.55 million nurses/midwives to the total shortages by 2030);^^^includes seat expansion by 22 seats per institution;## Annual shortages estimated including 0.51 AYUSH practitioners to the total shortages by 2030.
