# Supplementary material for: Achieving universal health coverage and sustainable development goals by 2030: investment estimates to increase production of health professionals in India
Source: Hum Resour Health. 2023 Mar 2;21:17. doi: 10.1186/s12960-023-00802-y (PMC9979880; doi:10.1186/s12960-023-00802-y)
Supplement: Supplementary file 2 — Additional file 2. Estimation of Investment Need for Increased Production of Health Workforce. [file 12960_2023_802_MOESM2_ESM.docx]

**Additional file 2**

**Estimation of Investment Need for Increased Production of Health Workforce**

*Baseline numbers, 2020*

Baseline numbers were arrived at by linearly projecting the total production and stock numbers as of 2020 (Equations (1) and (2)). For active health workforce, we applied the worker population ratio (WPR) for doctors and nurses(/midwives) separately as estimated from PLFS 2018-19 on the projected population as of January 2020 (Equation (3)). For projection of total production and stock of health professionals and active health workforce up to 2030 we used standard method as discussed in Ridoutt et al. (1). However, we used a range of indicators from India to modify the method for the present analysis purpose.

$Total production \left( P_{h2020} \right)={NHWA}_{h2018}+(s_{h2014}+s_{h2015})$ ………………………………… (1)

Where ‘$P_{h2020}$’ is total production of health professionals in year 2020. ${NHWA}_{h2018}$ estimated from NHWA 2018 data. Subscript ‘h’ stands for types of health workers (doctors and nurses(/midwives)). NHWA is NHWA data and ‘s’ is total annual admission of medical students across all institutions in India.

$S_{h2020}=P_{h2020}-(M_{h2020}+D_{h2020}+R_{h2020})$ …………………………………………………........ (2)

Where, S is total stock of health professionals, $M_{h2020}, D_{h2020} and R_{h2020}$ are total net migration, deaths and retirement in the year 2020.

${HW}_{h2020}={WPR}_{h2019}*popl2020$…………………………………………………………………………….. (3)

Where HW is the active health workforce, WPR is worker population ratio estimated from PLFS 2018-19 for respective categories of health workers, ‘popl2020’ is projected population as of January 2020.

*Projections up to 2030*

Total production of health professionals was estimated for each of the year between 2020 and 2030 using the baseline number as presented in equation (1) and annual admission of students in all medical institutions with a five years lag (equation 4). We included estimated pass-outs from all new institutions established or announced to be established up to 2025.

$P_{ht}=P_{h2020}+ s_{ht-5}$ …………………………………………………………………………………………………. (4)

Where subscript ‘t’ is year of estimation.

Accordingly, total stock of health professionals and active health workers were estimated using equations (5) and (6) respectively, as follows:

$S_{ht}=P_{ht}-(M_{ht}+D_{ht}+R_{ht})$ ………………………………………………………………………………….. (5)

We used a constant net migration and retirement rates for future years as estimated for the year 2020 and allowed a declining trend of death rates from current level of 2.5 to 2.1 up to 2030

${HW}_{ht}=(S_{h2020}-\left( {OW}_{2019}+U_{2019}+{NW}_{2019} \right))+ {(s}_{ht-5}-({OW}_{ht}+U_{ht}+{NW}_{ht}))$……………………………………………………………………….………………………………………………... (6)

Where OW is health professionals out of labourforce, U is unemployed, and NW is health professionals working in non-human health services. OW, U and NW were estimated from PLFS 2018-19 and was used for the future years up to 2030. We marginally moderated (downward) these rates for the future years with the assumption that these rates may decline in future with growing demand of health workers in general.

*Needs and shortages estimation in HRH, 2020 to 2030*

In this study, we have used International Labour Organization (ILO) & World Health Organization (WHO) recommended (2,3,4–8) HRH density threshold, 34.5 and 44.5 skilled health worker per 10,000 population respectively, to achieve SDGs (2,3,4–8), for estimating the actual HRH need, shortages and also for estimating the quantum of investment required to meet these HRH thresholds. We have considered doctor: nurse(/midwives) ratio of 1:2 within the overall recommended threshold and using population projection, we estimated the total need and the gaps in stock and active health workforce, segregated by the required number of doctors and nurses(/midwives) respectively.

*Strategies and required investment for overcoming the projected HRH shortages, by 2030*

The proposed strategies to increase production of health professionals and the required investment to bridge the existing and potential gaps in HRH by 2030 are based on alternative scenarios of a combination of increased utilisation (pass-out from existing institutions) of existing seats in nursing institutions, intake capacity (expansion of seats), opening of new medical and nursing colleges and encouraging out-of-workforce health professionals to join health workforce (Table 1).

The required investment was estimated by multiplying the unit cost of opening new institutions and/or expanding the existing seat capacity of institutions by number of institutions/seats required. We estimated unit costs of opening new institutions by dividing total government budget allocation for opening new institutions by number of new institutions separately for medical colleges and nursing institutions (9,10). Unit cost of seat expansion was estimated as total budgetary allocations in existing colleges divided by total number of seats in those colleges (11,12,13). The estimated unit costs were further discussed with experts and government personnel and were adjusted for including private sector institutions.

The proposed different scenarios represent different bounds of investments: 1) lower bound, to overcome the projected shortages in total stock 2) upper bound, to overcome the projected shortages in active health workforce. and 3) middle bound, by reducing at least 50% of the existing labour market attrition by 2030. Two alternative scenarios are also presented as i) if all proposed seat expansion is considered only in government institutions and ii) if indigenous medicine (*Ayurveda, Yoga, Unani, Siddha and Homeopathy* [AYUSH]) practitioners considered as a part of total health workforce.

*Benefits of investment in HRH*

Although investments in health, including health workforce, have multiple benefits within and beyond health sector, we only estimated a limited benefit of such investment in terms of employment generation (eq. 7) and its contribution to national income (eq. 8). Total additional employment for the year 2030 was estimated by adding potential additional employment of ‘support staff’ and ‘health associates’ based on previous study estimates (14). The health associates include dieticians and nutritionists, optometrists and opticians, dental assistants, physiotherapy associates, pharmacist assistants, occupational therapist and so on. The support staff includes ambulance drivers, professionals, garbage collectors, cashiers, clerks, mechanics, finance personnel and others (14).

*Increased Employment*

${\Delta E}_{h2030}={\Delta HW}_{h2030}+{\Delta SS}_{h2030}+{\Delta HA}_{h2030}$………………………………………………………. (7)

Where, ${\Delta E}_{h2030}$ is additional number of all workers employed in health sector by the year 2030 $, {\Delta HW}_{h2030}, {\Delta SS}_{h2030} and {\Delta HA}_{h2030}$ are additional health workforce (doctors and nurses(/midwives)), support staff and health associates respectively in the year 2030 due to the enhanced investment.

*Contribution to national income*

The labour productivity in health sector for the year 2019 was estimated as follows:

${{LP}_{h2019}=GVA}_{h2019}/E_{h2019}$…………………………………………………………………………………. (8)

Where ${LP}_{h2019}$ is labour productivity of health sector workers and ${GVA}_{h2019}$is gross value added in health sector in 2019 at current prices.

Finally, total benefits of enhanced investment in health workforce was estimated as total contribution to gross value added by new employment because of the enhanced investment as follows:

${\Delta GVA}_{h2030}=\Delta E_{h2030}*{LP}_{h2019}$ …………………………………………………………………………. (9)

Where ${\Delta GVA}_{h2030}$ is changes in gross value added in health sector and ${\Delta E}_{h2030}$is additional employment because of the enhanced investment.

**REFERENCES**

1. Ridoutt L, Cowles C, Madden L, Stewart G. Planned and unplanned futures for the Public Health Physician Workforce in Australia. Sydney. 2017;127.
2. Campbell J, Dussault G, Buchan J, Pozo-Martin F, Guerra Arias M, Leone C, et al. A universal truth: no health without a workforce. Forum report, third Global Forum on Human Resources for Health, Recife, Brazil. Geneva: Global Health Workforce Alliance and World Health Organization;2013. <https://www.who.int/workforcealliance/knowledge/resources/GHWA_AUniversalTruthReport.pdf> (Accessed 13 Dec 2020.)
3. WHO. Global strategy on human resources for health: workforce 2030. Geneva, World Health Organization. 2016. <https://apps.who.int/iris/bitstream/handle/10665/250368/9789241511131-eng.pdf?sequence=1>. (Accessed 13 Dec 2020).
4. Social Protection Floors Recommendation (202), 2012: National Floors of Social Protection. Geneva, International Labour Office, 2012.
5. Social health protection: an ILO strategy towards universal access to health care. Geneva, International Labour Organization, 2008 Social Security Policy Briefings; <https://www.ilo.org/secsoc/information-resources/publications-and-tools/policy-papers/WCMS_SECSOC_5956/lang--en/index.htm> (Accessed 12 Jun 2021).
6. World Social Security Report 2010–2011: providing coverage in times of crisis and beyond. Geneva, International Labour Office, 2011.
7. Scheil-Adlung X et al. New approaches to measuring deficits in social health protection coverage in vulnerable countries. Geneva, World Health Organization, 2010 <https://www.who.int/healthsystems/topics/financing/healthreport/BP56MeasurementILO.pdf> (Accessed 12 Jun 2021).
8. Scheil-Adlung X. Health workforce benchmarks for universal health coverage and sustainable development. Bull World Health Organization ;91(11):888–9.<https://www.who.int/bulletin/volumes/91/11/13-126953.pdf> (Accessed 12 Jun 2021).
9. Lok Sabha, Ulaka SS, Jadhav P, Chaudhary P, Bohra R. New medical colleges. Government of India. Lok Sabha, Ministry of Health And Family Welfare, Department Of Health And Family Welfare;(113):6. <http://164.100.24.220/loksabhaquestions/annex/174/AU1090.pdf>. (Accessed 28 Jan 2021)
10. Bihar Medical Services & Infrastructure Corporation. Medical infrastructure uploads, JAN-18. Bihar Medical Services & Infrastructure Corporation, Government of Bihar, India. 2018. <http://bmsicl.gov.in/uploads/Infrastructure/JAN-18.pdf>. (Accessed 6 Mar 2021).
11. Ministry of Health and Family Welfare. Medical Education Policy & Medical Education, 14 Chapter, document, 2018-19. Ministry of Health and Family Welfare, Government of India, 2019. <https://main.mohfw.gov.in/sites/default/files/14%20Chapter%20237-239AN2018-19.pdf>
12. Sharma G. GoI approves upgradation of 3 more Nursing Schools into Colleges. Daily Excelsior. 2020.<https://www.dailyexcelsior.com/goi-approves-upgradation-of-3-more-nursing-schools-into-colleges/> (Accessed 6 Mar 2021).
13. Government of India. Status of implementation of various schemes under strengthening/upgradation of Nursing Services (Human Resource for Health) and Development of Nursing services during XIth Plan. 2014. <http://nursingandmidwifery.gov.in/11thFiveyearPlan.pdf>. (Accessed 6 Mar 2021)
14. Karan A, Negandhi H, Nair R, Sharma A, Tiwari R, Zodpey S. Size, composition and distribution of human resource for health in India: new estimates using National Sample Survey and Registry data. BMJ Open. 2019;9(4):e025979.[https://doi.org/10. 1136/bmjopen-2018-025979](https://doi.org/10.%201136/bmjopen-2018-025979). (Accessed on 3 Apr 2021).
